# Precise and interpretable neural networks reveal epigenetic signatures of aging across youth in health and disease

**DOI:** 10.3389/fragi.2024.1526146

**Published:** 2025-01-23

**Authors:** David Martínez-Enguita, Thomas Hillerton, Julia Åkesson, Daniel Kling, Maria Lerm, Mika Gustafsson

**Affiliations:** ^1^ Division of Bioinformatics, Department of Physics, Chemistry and Biology, Linköping University, Linköping, Sweden; ^2^ Department of Forensic Genetics and Toxicology, Swedish National Board of Forensic Medicine, Linköping, Sweden; ^3^ Division of Inflammation and Infection, Department of Biomedical and Clinical Sciences, Linköping University, Linköping, Sweden

**Keywords:** DNA methylation, neural networks, age clock, epigenetic age, youth

## Abstract

**Introduction:**

DNA methylation (DNAm) age clocks are powerful tools for measuring biological age, providing insights into aging risks and outcomes beyond chronological age. While traditional models are effective, their interpretability is limited by their dependence on small and potentially stochastic sets of CpG sites. Here, we propose that the reliability of DNAm age clocks should stem from their capacity to detect comprehensive and targeted aging signatures.

**Methods:**

We compiled publicly available DNAm whole-blood samples (n = 17,726) comprising the entire human lifespan (0–112 years). We used a pre-trained network-coherent autoencoder (NCAE) to compress DNAm data into embeddings, with which we trained interpretable neural network epigenetic clocks. We then retrieved their age-specific epigenetic signatures of aging and examined their functional enrichments in age-associated biological processes.

**Results:**

We introduce NCAE-CombClock, a novel highly precise (R^2^ = 0.978, mean absolute error = 1.96 years) deep neural network age clock integrating data-driven DNAm embeddings and established CpG age markers. Additionally, we developed a suite of interpretable NCAE-Age neural network classifiers tailored for adolescence and young adulthood. These clocks can accurately classify individuals at critical developmental ages in youth (AUROC = 0.953, 0.972, and 0.927, for 15, 18, and 21 years) and capture fine-grained, single-year DNAm signatures of aging that are enriched in biological processes associated with anatomic and neuronal development, immunoregulation, and metabolism. We showcased the practical applicability of this approach by identifying candidate mechanisms underlying the altered pace of aging observed in pediatric Crohn’s disease.

**Discussion:**

In this study, we present a deep neural network epigenetic clock, named NCAE-CombClock, that improves age prediction accuracy in large datasets, and a suite of explainable neural network clocks for robust age classification across youth. Our models offer broad applications in personalized medicine and aging research, providing a valuable resource for interpreting aging trajectories in health and disease.

## Introduction

Age estimation through DNA methylation (DNAm) age clocks has been a considerably successful application of epigenetic data for modeling purposes. A wide variety of these clocks have been developed, differing in their training approaches, sources of tissue, and study populations (Horvath, 2013; Horvath et al., 2018; Hannum et al., 2013; Levine et al., 2018; Zhang et al., 2019; Li et al., 2018; Aanes et al., 2023)⁠, even including non-human species (Lu et al., 2023)⁠. First-generation DNAm clocks aim to accurately track chronological age by leveraging variations in the methylation levels of specific genomic locations (CpG sites) that occur throughout lifetime (Zavala et al., 2024)⁠, whereas second-generation age clocks incorporate other biological and clinical parameters to predict all-cause mortality risk (McCrory et al., 2021; Liu et al., 2018; Levine et al., 2018)⁠. The divergence between estimated and true chronological age is called epigenetic age acceleration, with a positive value indicating a faster rate of biological aging. An accelerated aging pace has been linked to an increased risk of cardiovascular, musculoskeletal, and neurodegenerative disorders (Chen et al., 2016; Levine, 2013; Levine et al., 2018)⁠ and general adverse health outcomes, such as frailty, cognitive decline, or immune dysregulation (Belsky et al., 2015; Elliott et al., 2021)⁠. However, while changes that slow epigenetic aging often correlate with increased longevity, recent research demonstrated that interventions extending cellular lifespan do not consistently affect epigenetic aging rates (Kabacik et al., 2022)⁠. Altogether, this emphasizes the premise that aging is the distinct, deterministic, and malleable consequence of genetic, environmental, and stochastic mechanisms operating and interacting in parallel (Rando and Wyss-Coray, 2021; Tong et al., 2024)⁠.

The importance of precise age estimation is particularly pronounced in adolescents and young adults due to the profound biological changes occurring during these stages, which shape the future health trajectory of an individual. During childhood and adolescence, DNAm patterns undergo significant modifications, accelerating epigenetic aging as part of natural development (Simpkin et al., 2017; Jones et al., 2015)⁠. These changes can be further influenced by adverse conditions such as disease, physical or emotional abuse, familial instability, and economic hardship, which can expedite the aging process (Marini et al., 2020; Sumner et al., 2023)⁠. Consequently, providing accurate age estimates for individuals in their adolescence and young adulthood is highly relevant in developmental healthcare, forensic science, criminology, and the study of population dynamics (Bozack et al., 2023; Vidaki and Kayser, 2017; Hjern et al., 2012)⁠. DNAm age clocks can help identify individuals with developmental disorders or those at risk of accelerated aging due to environmental stressors, enabling timely interventions to mitigate these effects. They can also provide assistance in legal and criminal proceedings and inform public health strategies and policies about the aging patterns of populations exposed to different socioeconomic conditions. This information may allow for the adequate allocation of resources, infrastructure, and services to improve the wellbeing of these populations.

Despite the accuracy of existing DNAm age clocks, their role as predictors of biological aging or lifespan is hindered by their limited interpretability, even when trained using extensive clinical and omics data (Fong et al., 2024)⁠. Current age clocks rely on disparate sets of CpG sites with minimal overlap and ambiguous association with risk factors of aging, which has led to concerns about their signal-to-noise ratios (Oblak et al., 2021; Liu et al., 2020; Higgins-Chen et al., 2022)⁠. The question has been raised of whether the precise estimates achieved by these clocks primarily reflect stochastic deviations of DNAm levels rather than true biological aging processes (Tong et al., 2024; Zhang et al., 2019; Tarkhov et al., 2024; Seale et al., 2022)⁠. Recent studies suggest that, while epigenetic age can be accurately modeled as the cumulative variation across methylation sites (Meyer and Schumacher, 2024), changes in DNAm patterns at age-linked CpGs extend beyond mere stochastic fluctuations, showing significant associations with disease phenotypes (Dabrowski et al., 2024)⁠. Traditional CpG site selection methods, such as elastic net, by definition omit large portions of the epigenome. These approaches can be advantageous as increasing the number of included sites yields diminishing returns in age clock performance (Dabrowski et al., 2024)⁠. However, they also risk excluding aging markers that could hold relevant biological insights. Other approaches use principal components of DNAm data and have demonstrated improved reliability (Fong et al., 2024; Higgins-Chen et al., 2022),⁠ but they are fundamentally constrained to modeling linear effects and may fail to capture the complex, interconnected nature of aging mechanisms. Instead, incorporating data from diverse, well-annotated datasets within a non-linear framework, such as neural network embeddings, may enhance the generalizability and robustness of age clocks by better capturing subtle patterns in DNAm data.

Here, we hypothesized that the reliability of an epigenetic age estimation model should be grounded both in its accuracy and its capacity to detect comprehensive and targeted DNAm signatures linked to physical and psychological developmental mechanisms associated with aging. We propose that leveraging a compressed representation of DNAm data as input for explainable neural network models, such as embeddings from a pre-trained autoencoder, may provide an adequate balance between precision and efficiency. Employing interpretable data-driven methods that utilize markers across the measurable human methylome ensures that the model serves not only as a predictive tool but also as a valuable informative resource, facilitating the understanding of the biology behind epigenetic age prediction. Our approach aims to improve the robustness of age estimation by capturing fine-grained signatures of age-related DNAm variation throughout the human lifespan, with a particular focus on critical developmental stages such as adolescence and young adulthood, at single-year resolution. In summary, we seek to uncover the underlying biological processes driving age-related health trajectories and vulnerabilities, thereby advancing the current knowledge on human aging and paving the way for future applications at the clinical level.

## Materials and methods

### Data collection and preprocessing

The data used in this study consisted of publicly available DNA methylation (DNAm) samples from individuals with reported age from the Gene Expression Omnibus (GEO) repository (n = 30,228) from either the Illumina Infinium HumanMethylation450K or MethylationEPIC BeadChip arrays. DNAm beta values were normalized using Gaussian mixture quantile normalization (GMQN) (Xiong et al., 2022)⁠. Low-quality samples were filtered out using the ChAMP R package (v2.30.0), and missing beta values were imputed using k-nearest neighbors (k = 10) from the bnstruct R package (v1.0.15). Non-CpG probes, probes related to single-nucleotide polymorphisms (SNPs), multi-hit probes, and probes not shared by both Illumina 450K and EPIC arrays were removed. Probes located on the X and Y chromosomes were kept only for XY models. In total, 384,629 CpG sites were left after pre-processing or 395,248 CpG sites for XY models.

DNAm samples were split into training and test sets according to their application. Age regression performance was evaluated using 17,726 whole-blood samples (0–112 years, mean ± SD age = 41.0 ± 22.9 years, and 45.6% female). DNAm samples from various other tissues were used to demonstrate the model’s applicability across different contexts, including peripheral blood lymphocytes (PBLs), peripheral blood mononuclear cells (PBMCs), buccal epithelium, saliva, muscle, and semen (Supplementary Tables S1, S2). Whole-blood samples were divided into a young-age set (10–30 years, n = 1,404, mean ± SD age = 21.8 ± 5.3 years, and 46.5% female) and a complete-age set (1–101 years, n = 8,846, mean ± SD age = 48.6 ± 19.4 years, and 45.2% female) to train and benchmark the classification performance of DNAm age clocks with 15-, 18-, and 21-year cutoffs. In addition, a sex-balanced set (14–24 years, n = 330, mean ± SD age = 19.1 ± 3.1 years, and 48.7% female) formed by 11 single-age cohorts of 30 healthy controls was used to evaluate classifiers at specific ages. To investigate epigenetic aging in adolescents and young adults with Crohn’s disease (CD), we compiled five whole-blood DNAm datasets (GSE112611, GSE32148, GSE81961, GSE87640, and GSE87648) and selected individuals between 14 and 24 years, resulting in 207 treatment-naive CD patients (mean ± SD age = 17.1 ± 2.5 years and 38.2% female) and 52 healthy controls (mean ± SD age = 18.6 ± 3.5 years and 50.0% female). A detailed summary of all samples used is available in Supplementary Material S1.

### Design and training of neural network models for age estimation

Artificial neural network models were trained using the Keras 2.4.3 library with TensorFlow 2.4.0 and TensorFlow-GPU 2.2.0 backend, implemented for Python 3.8.10. The deep methylation network-coherent autoencoder (NCAE) used to compress DNAm samples into embeddings was trained as described in Martínez-Enguita et al. (2023). It consisted in a three-hidden layer model with a width of 128 hidden nodes per layer, with a leaky rectified linear unit (leaky ReLU, α = 0.3) as the activation function and a sigmoid output layer function, and with mean squared error (MSE) as the loss function. Embeddings were retrieved from the 128-dimensional latent space of the third hidden layer. Supervised deep neural network (DNN) models for age estimation using NCAE embeddings (referred to as NCAE-Age models) are feed-forward, fully connected, three-layered DNNs with either an age regression (prediction of a sample’s age in years) or a binary classification (probability for the age of a sample to be equal or above a certain cutoff) training objective. The target age values [0, 114] were min–max scaled to promote the numerical stability and enhance model performance. An NCAE leveraging CpG sites from the X and Y chromosomes as additional input features (NCAE-XY) was used to obtain DNAm embeddings to train NCAE-XY-Age DNN models, with the same architecture and training strategy as described.

NCAE-Age regressors were trained using the Adam optimizer to minimize the MSE (learning rate = 1e-4, β1 = 0.9, β2 = 0.999, ε = 1e-7, decay = 1e-6), with leaky ReLU (α = 0.3) as the hidden layer activation, to prevent the “dying ReLU” problem, and output layer function, He uniform initializer, L1 kernel regularization (λ = 0.01) on the third hidden layer, dropout (p = 0.1) and batch normalization (momentum = 0.99, ε = 1e-3) on every hidden layer, and batch sizes of 64 to 1,024. Binary classifiers were trained on the young-age set, with cutoffs of 12–26 years, using the Adam optimizer to minimize the binary cross-entropy (same parameters as previously mentioned) with an exponential decay learning rate schedule (rate = 0.95, 1e3 steps), leaky ReLU (α = 0.3) as hidden layer activation function, sigmoid output layer, He uniform initializer, L2 kernel regularization (λ = 1e-3), dropout (*p* = 0.1) and batch normalization (momentum = 0.99, ε = 1e-3) on every hidden layer, and batch size of 32. Sample weights were introduced during training to compensate for class imbalances. DNNs were trained until early stopping (patience = 1e3 epochs) using an 80:20 ratio of training and validation, respectively.

### Selection and benchmarking of DNA methylation age clocks

Numerous DNAm age clocks have been developed, differing on training data, methodology, assumptions, and selection of CpG sites. We conducted a literature search to find epigenetic clocks that met the following criteria: the clock should be (a) trained and evaluated using DNAm data (b) from whole-blood samples, using publicly available (c) methodology and (d) a set of CpG sites to (e) estimate the chronological age and provide measurements of biological aging (age acceleration). We benchmarked NCAE-Age models and other relevant DNAm age clocks: Horvath pan-tissue (Horvath, 2013)⁠, Horvath skin and blood (Horvath et al., 2018)⁠, Hannum (Hannum et al., 2013)⁠, PhenoAge (Levine et al., 2018)⁠, Zhang (elastic net) (Zhang et al., 2019)⁠, Li (Li et al., 2018)⁠, and PAYA (Predictor for Adolescents and Young Adults) (Aanes et al., 2023)⁠. The CpG sites and reported coefficients of the selected DNAm age clocks are available in Supplementary Material S2. In addition to them, we included a consensus model (“top-five”) based on the median prediction of the five best models from each evaluation; CombClock, a novel age-regressor trained using elastic net (α = 0.5) on the combined 1,743 CpG sites of the selected clocks; and NCAE-CombClock, a supervised DNN utilizing the 1,743 CpG sites plus 128 NCAE embeddings, with the same architecture and training strategy as described for NCAE-Age regressors.

The regression benchmark was conducted using 17,726 samples from healthy individuals (0–112 years) divided into training and test sets (80:20). Retraining of elastic net age clocks was performed as described in the classification benchmark. The performance was measured using the coefficient of determination (R^2^), mean absolute error (MAE), median absolute error (MedAE), root-mean-square error (RMSE), Pearson correlation coefficient (PCC, *r*), and Spearman correlation coefficient (ρ). Performance metrics were calculated using the MLmetrics R package (v1.1.1).

The classification benchmark was conducted using the young-age (10–30 years, n = 1,404) and complete-age (1–101 years, n = 8,846) sets. Both sets were used to retrain the DNAm age clocks to account for differences in data and pre-processing between study settings. The retraining was performed by reimplementing the elastic net regression (α = 0.5) using the glmnet R package (v4.1-8). An intercept was included if present originally. In the young-age setting, a five-fold cross-validation approach was applied to train and evaluate the age clocks. Each model was trained in four folds, while the left-out fold was used as the test set, until every fold was circulated. In the complete-age setting, models were trained in four folds of young-age samples plus all complete-age samples. They were evaluated on the left-out young-age fold, again until every fold was evaluated. DNAm age clocks were tested on their capacity to predict whether a sample belonged to an individual equal to or over or under the cutoffs of 15, 18, and 21 years. To obtain a comparable metric between DNAm age clocks, estimated ages were binarized based on the cutoffs. The performance was measured using the F1-score, the false-positive rate (FPR) above and below cutoffs, and the total for each fold. Since NCAE-Age classifiers generate probability estimates, unlike other DNAm age clocks, their performance was also evaluated using the area under the receiver operating characteristic curve (AUROC). Accuracy and probability estimates are reported for classifiers on single-age cohorts from the balanced test set.

### Identification of DNA methylation signatures of aging

Light-up analyses were performed to retrieve importance rankings of the input features (CpGs) from the trained NCAE-Age model. The light-up technique (Dwivedi et al., 2020; Martínez-Enguita et al., 2023)⁠ for the interpretation of supervised DNNs is based on the recursive forward propagation of perturbations on the input (in this case, CpG sites, either toward hypermethylation, β = 1, or hypomethylation, β = 0) of pre-trained models (in this case, a concatenated NCAE and NCAE-Age model). Input samples for light-up may consist of DNAm profiles representative of a specific cohort of interest, e.g., 21-year-old healthy controls. Perturbations are added iteratively to input CpGs and propagated through the concatenated model layers. The contribution of a CpG to the training objective is measured by the observed changes (delta values, δ) in the model outcome, i.e., changes in the predicted epigenetic age of the sample. Then, δ-values are used to rank the CpG input list by relevance to the model’s training objective. We selected the top 1,000 CpGs in increasing order of negative light-up δ-value, which we refer to as the NCAE-Age or NCAE-XY-Age DNAm signature of aging for a particular age estimation DNN and a particular condition and age, for further analysis.

### CpG annotation and functional enrichment analysis

Genome-wide mapping of CpG probes to genes was performed using Infinium MethylationEPIC probe annotation files from ChAMP (v2.30.0) and ChAMPdata (v2.32.0) R packages. Gene symbols were annotated to Entrez or NCBI (National Center for Biotechnology Information) gene IDs using AnnotationDbi (v1.62.2) and org.Hs.eg.db (v3.17.0) R packages. Functional gene enrichment analysis of CpG-associated DNAm signatures in Gene Ontology (GO) terms from the “Biological Process” category was performed using the clusterProfiler R package (v4.8.3) with default parameters and FDR adjustment. GO terms with FDR-adj. p < 0.05 were considered significantly enriched and were grouped into manually curated ancestor term categories using annotations from QuickGO (https://www.ebi.ac.uk/QuickGO/ Accessed on 30 May 2024). GO terms displayed in heatmaps are clustered by the z-score of their gene ratios across significant enrichments for each case, including the mean and standard deviation of the gene ratios.

### Statistical analyses and visualization

Statistical analyses and data processing were conducted in R 4.4.0, within RStudio 2023.12.1, and Python 3.8.10. Confidence intervals are 95% CIs, unless otherwise specified. Wilson score intervals were employed to calculate CIs for classifier accuracy to account for moderate cohort sizes and accuracy values close to the upper bound. Figures were created using ggplot2 (v3.4.4), gplots (v3.1.3.1), corrplot (v0.92), and UpSetR (v1.4.0) R packages and Seaborn (v0.11.1) and matplotlib (v3.4.2) Python libraries. Subsequent figure editing for enhanced visualization was carried out in Inkscape v0.92.

## Results

### A deep neural network leveraging DNA methylation embeddings and CpG sites yields highly accurate age estimates

Initially, we evaluated the performance and generalizability of existing DNAm age clocks using large-scale datasets. We processed 17,726 publicly available whole-blood DNAm samples from individuals aged 0–112 years, with a mean ± SD age of 41.0 ± 22.9 years (Supplementary Material S1), and divided them into training and test sets (80:20). We then conducted a literature search for DNAm age clocks trained on whole-blood samples with a known set of CpG sites. We selected the clocks Horvath pan-tissue (Horvath, 2013)⁠, Horvath skin and blood (Horvath et al., 2018)⁠, Hannum (Hannum et al., 2013)⁠, PhenoAge (Levine et al., 2018)⁠, Zhang (elastic net) (Zhang et al., 2019)⁠, Li (Li et al., 2018)⁠, and PAYA (Aanes et al., 2023)⁠. These are based on elastic net and include 71–514 CpGs (Supplementary Material S2). Additionally, we included our NCAE-Age (Martínez-Enguita et al., 2023)⁠ model, a supervised deep neural network (DNN) age predictor that uses a compressed representation of the human methylome consisting of 128 variables (embeddings) from an autoencoder with a biologically relevant latent space, named network-coherent autoencoder (NCAE) (Martínez-Enguita et al., 2023)⁠. NCAE-Age clocks can be developed for multiple tissues or cell types, including whole blood, lymphocytes, buccal epithelial cells, and saliva (Supplementary Table S1).

**FIGURE 1:**
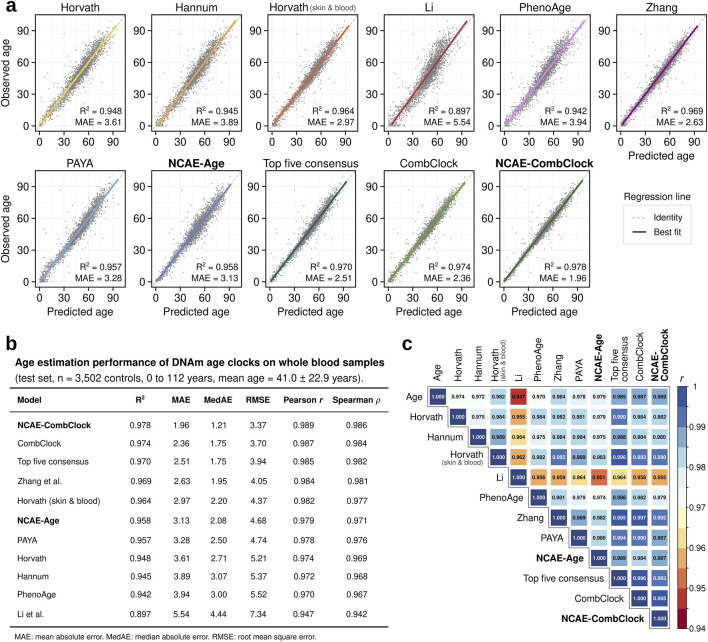
Regression performance benchmark of DNA methylation age clocks trained on whole-blood samples from healthy individuals. **(A)** Scatter plots comparing observed vs predicted ages for eleven DNAm age clocks on regression test set samples (n = 3,502 controls; 0–112 years). The coefficient of determination (R^2^) and mean absolute error (MAE) are indicated for each model. **(B)** Age estimation performance metrics for DNAm age clocks evaluated on the regression test set. **(C)** Correlation matrix of Pearson correlation coefficients (PCC) between the observed and predicted ages across DNAm age clocks.

We observed that the selected DNAm age clocks were able to deliver accurate estimates of chronological age (Figures 1A, B; Supplementary Material S3) for the test set samples (n = 3,502). Elastic net clocks achieved coefficient of determination (R^2^) values between 0.897 for Li’s clock (mean absolute error, MAE = 5.54 years; root-mean-squared error, RMSE = 7.34 years), optimized for age prediction in children and adolescents, to 0.969 for Zhang’s clock (MAE = 2.63 years; RMSE = 4.05 years), designed as a “near-perfect” age estimator across the entire age range. Horvath’s skin and blood clock performed better (R^2^ = 0.964, MAE = 2.97 years, and RMSE = 4.37 years) than its pan-tissue counterpart (R^2^ = 0.948, MAE = 3.61 years, and RMSE = 5.21 years). The NCAE-Age clock produced an R^2^ of 0.958 (MAE = 3.13 years and RMSE = 4.68 years), akin to PAYA (R^2^ = 0.957, MAE = 3.28 years, and RMSE = 4.74 years). Notably, we found that a consensus estimate based on the median of the predictions from the five top-performing clocks outperformed every other DNAm age clock tested, with an R^2^ of 0.970 (MAE = 2.51 years and RMSE = 3.94 years). Building on this wisdom-of-the-crowds approach, we combined the 1,743 unique CpG sites used by the selected elastic net clocks to train a novel elastic net regressor model, named CombClock. Reassuringly, CombClock performed with a top test R^2^ of 0.974, an MAE of 2.36 years, and RMSE of 3.70 years. We thus inquired whether additional performance could be gained by leveraging both CpG markers and NCAE embeddings. For this purpose, we developed a high-precision hybrid model, which we refer to as NCAE-CombClock, which was trained as a supervised DNN with CpG sites and embeddings as input features. NCAE-CombClock achieved the highest R^2^ = 0.978 of all benchmarked models, with an MAE of 1.96 years and RMSE of 3.37 years.

DNAm age clock estimations had pairwise Pearson correlation coefficients (PCC) between 0.947 and 0.997 (Figure 1C). CombClock and Zhang predictions were the most similar (PCC = 0.997) due to Zhang being the largest contributor of CpG sites to CombClock. Estimated ages from elastic net clocks were less correlated to NCAE-CombClock predictions than to CombClock ones. For example, NCAE-CombClock had a lower correlation with Zhang’s clock (PCC = 0.992) than with CombClock. Likewise, predictions from the DNN-based clocks NCAE-Age and NCAE-CombClock were highly correlated (PCC = 0.987). Estimates from NCAE-CombClock were the closest to the true ages (PCC = 0.989), followed by estimates from CombClock, Zhang, and Horvath skin and blood (PCC = 0.987, 0.984, and 0.982, respectively). In summary, we showed that the use of large-scale training sample sets coupled with the combination of knowledge-driven CpG sites and data-driven embeddings of DNAm data enables the development of highly precise neural network age regressors, such as NCAE-CombClock, which is able to outperform other whole-blood DNAm age clocks in terms of R^2^, MAE, and PCC.

### A consensus of DNAm age clocks optimizes threshold-based age determination of adolescents and young adults

Next, we investigated whether predicting chronological age with accuracy can be translated into correctly determining if an individual is below or above specific age thresholds. We established a classification benchmark for our DNAm age clocks, focusing on three critical points of adolescence and young adulthood: 15, 18, and 21 years. These thresholds mark key transitional stages of youth development with significant implications in legal, health, and socioeconomic contexts. Importantly, they allow assessment of the practical applicability of DNAm age clocks in real-world scenarios where precise age classification is crucial. We, thus, aimed to identify the most reliable model for accurately categorizing samples by these age thresholds.

Hence, we applied five-fold cross-validation to predict the age of a subset of 1,404 whole-blood samples from healthy individuals (10–30 years) with mean ± SD age of 21.8 ± 5.3 years (Supplementary Material S1). To mitigate the impact of the varied age ranges in the original training data, we retrained DNAm age clocks, if applicable, in two different settings: young-age (10–30 years) and complete-age (1–101 years) (Supplementary Material S1). In both cases, we predicted the ages of the left-out young-age fold until the entire set had been processed, binarizing the outcomes based on whether they were lower (0) or equal to or higher (1) than 15, 18, or 21 years. To address class imbalances, and since this approach produces class predictions rather than probability estimates, we evaluated the classification performance using the fold-averaged F1-score.

**FIGURE 2:**
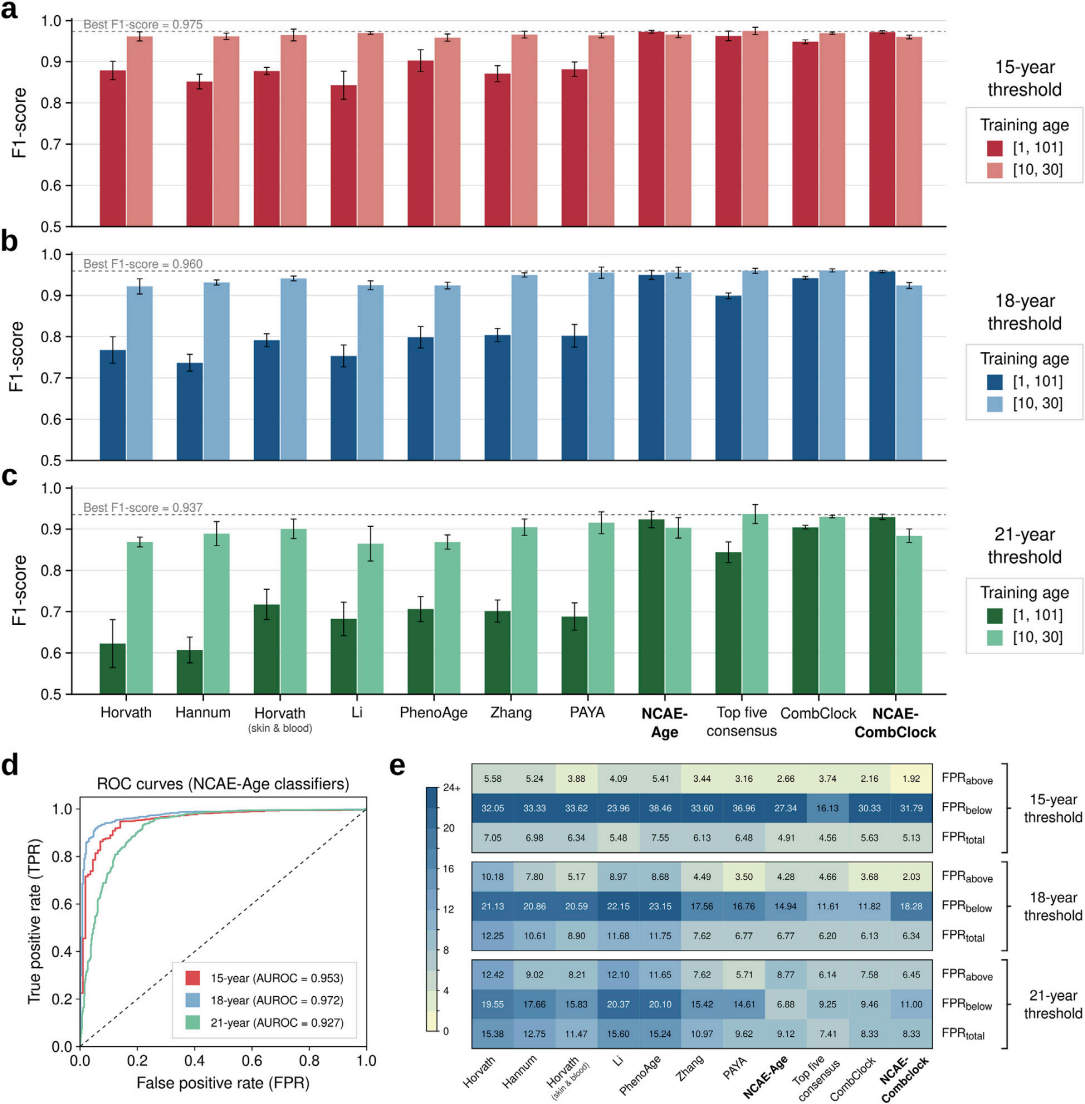
Classification performance benchmark of DNA methylation age clocks. **(A–C)** Bar plots of classification performance (F1-score) of DNAm age clocks based on their capacity to accurately determine if an individual’s chronological age is equal to or above the target age cutoffs of 15, 18, and 21 years. DNAm age clocks were evaluated on classification test set samples (n = 1,404 controls; 10–30 years) divided into five folds. Models were retrained, if applicable, in complete-age (1–101 years) or young-age (10–30 years) training settings. The best average F1-score per target age is indicated and marked as a gray dotted line. Error bars represent the 95% confidence interval. **(D)** Receiver operating characteristic (ROC) curves for NCAE-Age classifiers at 15, 18, and 21 years, evaluated on the classification test set. **(E)** Heatmaps of false positive rates (FPRs) for DNAm age clock estimations above or below the target age cutoffs of 15, 18, and 21 years and in total, considering the best-performing training setting in each case.

We found that the benchmarked DNAm age clocks can accurately classify samples based on specific young-age thresholds when adequately retrained (Figures 2A–C; Supplementary Material S3). Elastic net clocks were the most sensitive to the age range of the training set, underperforming on complete-age (F1-scores between 0.607 [95% CI: 0.568–0.646] and 0.903 [0.870–0.936]) compared to young-age (F1-scores between 0.865 [0.813–0.917] and 0.970 [0.966–0.974]). CombClock was only marginally affected when retrained on complete-age, decreasing in performance but still above 0.9 in all thresholds (F1-score = 0.948 [0.937–0.960] at 15 years, 0.942 [0.934–0.949] at 18 years, and 0.904 [0.893–0.915] at 21 years). DNN-based clocks NCAE-Age and NCAE-CombClock achieved better results in general on complete-age, with F1-scores between 0.924 [0.899–0.948] and 0.929 [0.914–0.945] at 21 years and 0.973 [0.969–0.977] and 0.972 [0.964–0.979] at 15 years. Overall, the classification performance expectedly decreased with a higher threshold age. The best clock was the young-age top five consensus, with the F1-score = 0.975 [0.964–0.986] for 15 years, 0.937 [0.908–0.966] for 21 years, and a tie with CombClock (0.960 [0.952–0.968] vs 0.960 [0.951–0.970] for CombClock) at 18 years. High performance in the regression benchmark was generally associated with strong performance in the classification benchmark, with significant correlations between R^2^ and F1-scores at the 18-year (PCC = 0.753, P = 7.43e-3) and 21-year (PCC = 0.793, P = 3.61e-3) thresholds, but not at 15 years (PCC = 0.229, P = 0.499). NCAE-Age clocks working as binary classifiers performed with area under the receiver-operating curve (AUROC) values of 0.953 for 15-year, 0.972 for 18-year, and 0.927 for 21-year thresholds (Figure 2D) under young-age settings. Regarding misclassification ratios measured at best-performing settings (Figure 2E), the top five consensus had the lowest total false-positive rate (FPR) at the 15-year (FPR_total_ = 4.56%) and 21-year (FPR_total_ = 7.41%) categories, while CombClock was marginally better at the 18-year category (FPR_total_ = 6.13% *versus* 6.20%). Most misclassified samples were due to underestimations of age, leading to high FPRs below the thresholds: 16.13%–38.46% (15-year), 11.61%–23.15% (18-year), and 6.88%–20.37% (21-year).

### An array of single-year NCAE-Age classifiers provides high-resolution young-age probability estimation

Our findings up to this point showed that DNAm age clocks can be optimized to serve as quasi-perfect predictors of chronological age, enabling accurate classifications of individuals based on thresholds of interest. However, even the top regressor (NCAE-CombClock) and the top age-classifier (top five consensus) exhibited non-negligible misclassification rates. Given the critical importance of precise age identifications in various real-world contexts, a more refined approach is required. We argued that an array of classifiers, each trained at a single-year cutoff, could collectively provide an ensemble of age probability estimates that might improve the robustness and reliability of age assessments at single-year categories.

**FIGURE 3:**
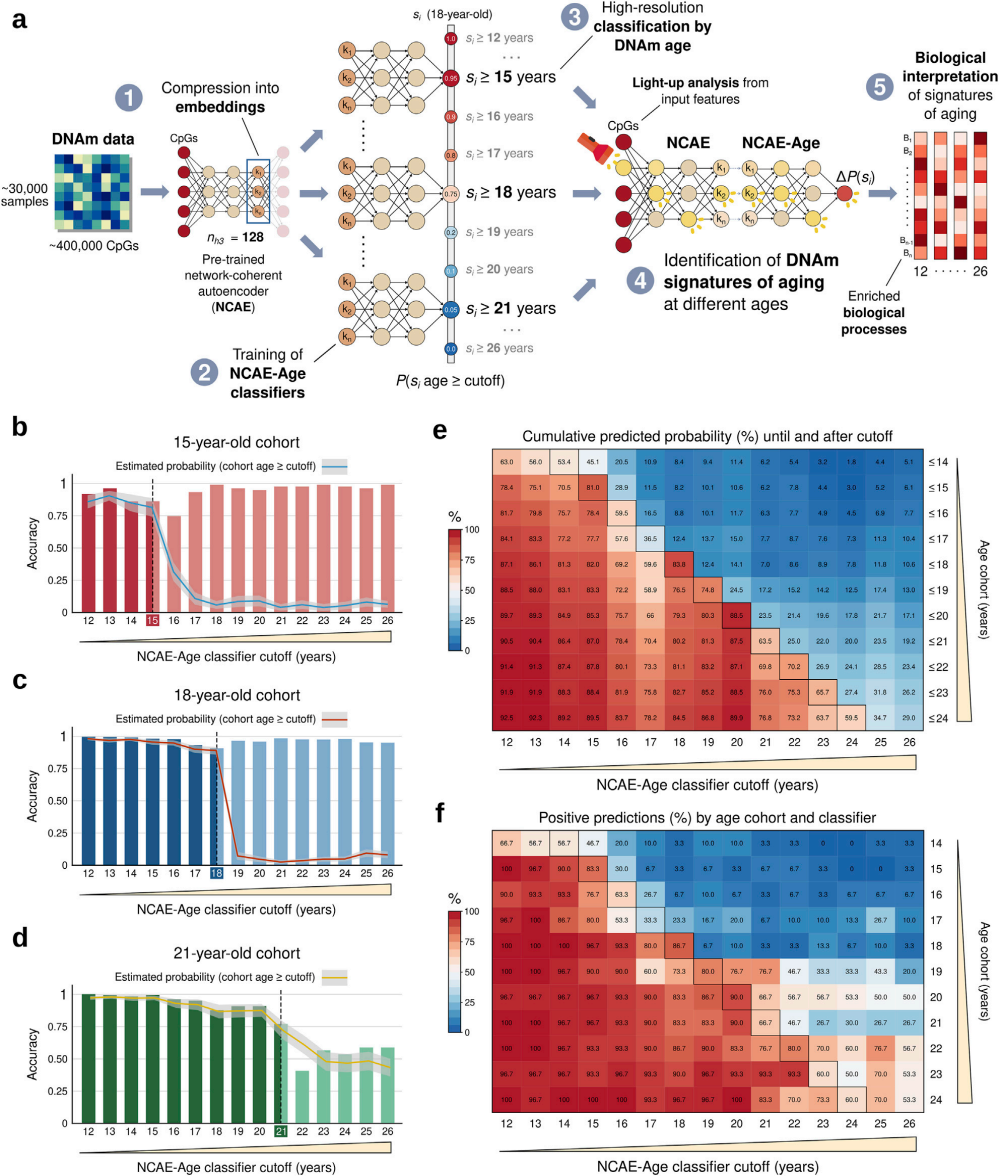
Workflow summary and performance evaluation of NCAE-Age classifiers by model cutoff and target age. **(A)** Training, evaluation, and biological interpretation of an array of NCAE-Age neural network classifiers from 128-dimensional network-coherent autoencoder (NCAE) embeddings of DNAm data. **(B–D)** Bar plots of binary classification accuracy of NCAE-Age classifiers for 15, 18, and 21 years. The overlaying lines indicate the probabilities estimated by each model for the cohort samples to be equal to or above the model cutoff (95% confidence interval). **(E)** Heatmap of cumulative predicted probabilities (%) for the balanced test set cohorts (n = 330; 14–24 years) to be equal to or above each NCAE-Age classifier cutoff, which were calculated until or after the cutoff. **(F)** Heatmap of positive predictions (%; estimated probability >0.5) per NCAE-Age classifier and balanced test set cohort. Outlined cells indicate that the true age of the test cohort is equal to the model cutoff. Age cohorts are sex-balanced groups of 30 controls of the same age, from left-out data.

Thus, we trained fifteen NCAE-Age classifiers with single-year cutoffs spanning through adolescence and young adulthood (12–26 years) using the young-age set (Figure 3A). We evaluated their classification performance using an age- and sex-balanced test set composed by eleven single-age cohorts of 30 controls (total n = 330, mean age ±SD = 19.1 ± 3.1 years, and 48.7% female) between 14 and 24 years. First, we applied the NCAE-Age array to generate probability estimates for DNAm samples from cohorts of the same age as the previous thresholds (15, 18, and 21 years) (Figures 3B–D; Supplementary Material S4). We determined a prediction to be positive if the classifier estimated a probability over 0.5 for a sample’s age to be equal to or above its target cutoff, and we calculated the accuracy per cutoff and cohort. For example, an accurate classifier with a cutoff of 14 years evaluating an 18-year-old sample should estimate a probability of at least over 0.5, indicating that it predicts that the sample’s age is above its cutoff. We found that all single classifiers achieved highly accurate classifications for the 15-year cohort (from 0.74, 95% CI [0.69–0.79] at the 16-year cutoff to 0.99 [0.97–0.99] at the 23- and 26-year cutoffs) and the 18-year cohort (from 0.90 [0.87–0.93] at the 18-year cutoff to 0.99 [0.97–1] at the 12-year cutoff), as well as for the 21-year cohort until the 21-year cutoff (from 0.77 [0.72–0.81] at the 21-year cutoff to 1 [0.99–1] at the 12-year cutoff). The accuracy decreased to between 0.40 [0.35–0.46] and 0.59 [0.53–0.64] when classifying 21-year-olds at cutoffs within the 22-to-26-year range. Expectedly, predicted probabilities sharply decreased when the age of cohort members became larger than the NCAE-Age cutoff. We also observed this decrease when considering the accumulated probabilities until or after each cutoff (Figure 3E), showcasing that the use of the complete classifier array leads to optimal classifications. By percentage of positive predictions (Figure 3F), 18-year-cutoff classifiers predicted 86.7% of 18-year samples to be equal to or above 18, down to 23.3% for 17-year samples, 6.7% for 16-year samples, and 3.3% for 15- and 14-year cohorts. Other NCAE-Age classifiers showing an above-average performance were the 15-year (83.3% for 15-year), 19-year (80.0% for 19-year cohort, only 6.7% for 18-year cohort), and the 22-year (80.0% for 22-year cohort) cutoffs. Cohorts between 20 and 24 years were harder to classify at the same granularity. Altogether, NCAE-Age classifiers enabled the precise categorization of individuals at single-year resolution, particularly accurately for cohorts from 14 to 19 years.

### Interpretable NCAE-Age clocks predict age by capturing developmental, immune, and metabolic mechanisms

We then analyzed the potential of the NCAE-Age classifier array as a data-driven tool capable of extracting meaningful epigenetic signatures of aging. We proposed that, for the estimates of a DNAm age clock to be a reliable reflection of the true epigenetic aging status of an individual, the model must effectively capture DNAm markers linked to age-associated biological mechanisms. By using interpretable models with specific age cutoffs that leverage a larger portion of the methylome, rather than fixed sets of CpGs, we could feasibly identify DNAm signatures describing aging processes throughout key development stages, such as adolescence and young adulthood, on a year-to-year basis.

**FIGURE 4:**
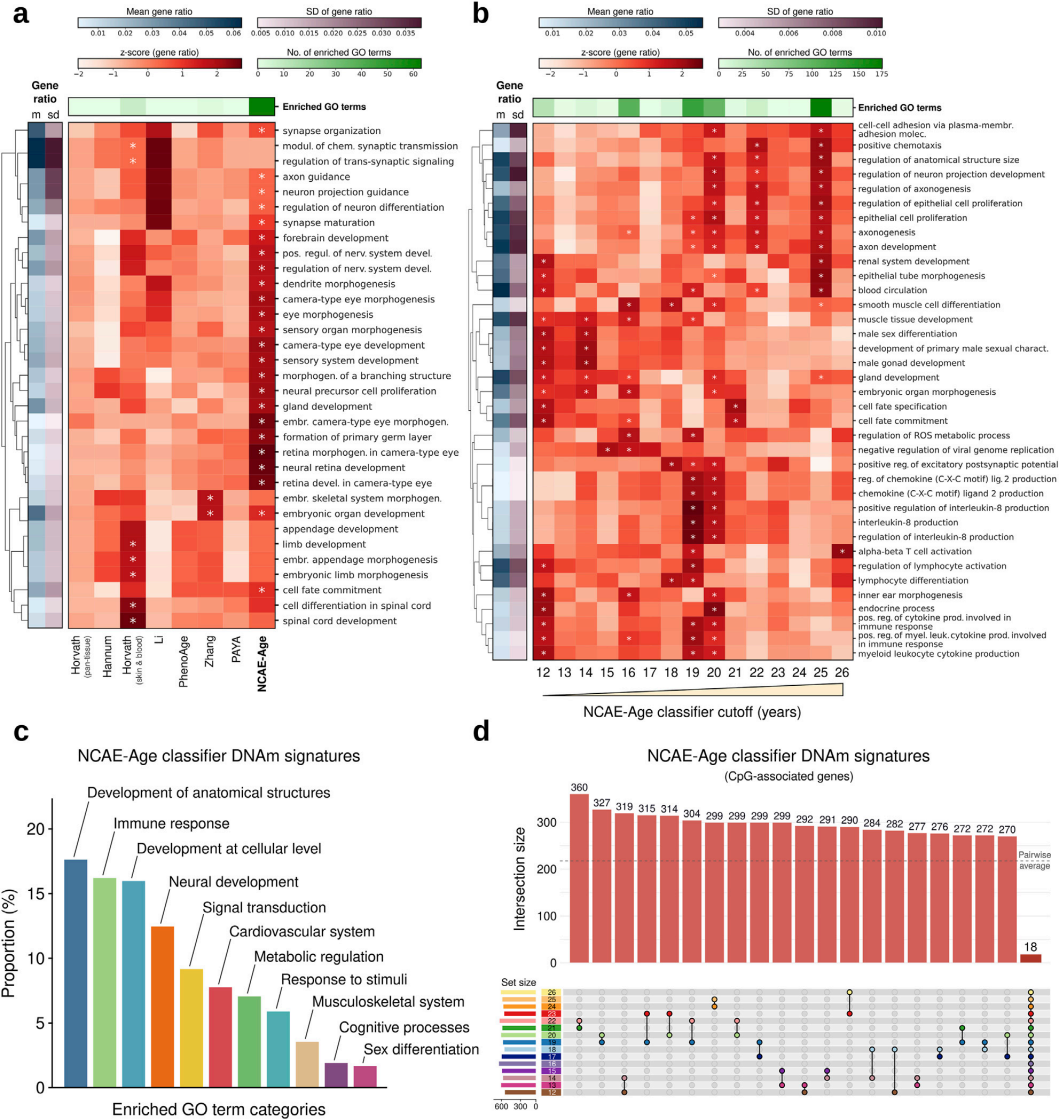
Functional enrichment analysis of CpG sites from DNAm age clocks and NCAE-Age DNAm signature genes. **(A, B)** Heatmaps of gene ratio z-scores of the top significantly enriched GO terms for CpGs from **(A)** DNAm age clocks and NCAE-Age DNAm aging signatures or **(B)** age-specific DNAm signatures of aging from NCAE-Age classifiers with cutoffs between 12 and 26 years. Significantly enriched (FDR-adj. p < 0.05) GO terms are marked with an asterisk (*). **(C)** Bar plot of the proportion (%) of significantly enriched GO terms (n = 426) across NCAE-Age DNAm signatures of aging and classifier cutoffs, grouped into common GO ancestor categories. **(D)** Upset plot of non-exclusive pairwise overlaps between CpG-associated genes from NCAE-Age classifier signatures of aging (12–26 years).

Thus, we first inspected the functional associations of the CpG sites used by DNAm age clocks and the epigenetic signatures from NCAE-Age models. We retrieved and annotated the CpG sets from age clocks (Supplementary Material S2). For NCAE-Age models, we applied the light-up technique (methods) to retrieve the contribution of CpG sites to the training objective of estimating age, selecting the top 1,000 as the DNAm signature of aging (Supplementary Material S5). We examined the biological context of the signature CpGs using Gene Ontology (GO) terms from the Biological Process category, finding significant enrichments (FDR-adj. P < 0.05) for CpGs from only Horvath skin and blood (eight GO terms) and Zhang (two GO terms) clocks (Figure 4A; Supplementary Material S7), with the combined 1,743 CpGs being enriched in 32 GO terms (Supplementary Material S7). By contrast, the DNAm signature of the NCAE-Age regressor was significantly enriched in 63 GO terms. Out of them, 31 (49.2%) were related with the morphogenesis and development of anatomical structures, such as the sensory, hepatic, and urinary systems, and 26 (41.3%) described the stages of neural system development, including the regulation of neurogenesis, synapse organization and maturation, and forebrain and telencephalon development. Other age-associated enriched GO terms were cell fate commitment and specification, locomotory behavior, response to reactive oxygen species, and regulation of signal transduction via small GTPases (Supplementary Material S7).

To increase the resolution of the analysis, we examined the DNAm signatures of aging from every NCAE-Age classifier from 12 to 26 years (Figure 4B; Supplementary Material S5), resulting in 426 significantly enriched GO terms across signatures (Supplementary Material S7). These included biological mechanisms associated with the development of anatomical structures (75/426; 17.6%) such as organ morphogenesis and growth regulation; immune response processes (69/426; 16.2%) such as leukocyte activation and cytokine production; developmental processes at the cellular level (68/426; 16.0%) such as regulation of cell fate specification, commitment, differentiation, and proliferation; neural development mechanisms (53/426; 12.4%) such as axonogenesis and synapse maturation; signal transduction pathways (39/426; 9.2%); and terms linked with the cardiovascular system (33/426; 7.7%), metabolic regulation (30/426; 7.0%), response to stimuli (25/426; 5.9%), musculoskeletal system (15/426; 3.5%), cognitive and behavioral processes (8/426; 1.9%), and sex differentiation (7/426; 1.6%) (Figure 4C). These signatures had a high overlap, but only 18 CpG-associated genes were shared among all of them (Figure 4D; Supplementary Material S5). Remarkably, we observed that, while enrichments in GO terms linked with development at both organismal and cellular levels were spread throughout the entire range, others were concentrated at specific ages. For example, DNAm signatures between 16 and 20 years were highly enriched in cognitive processes. Similarly, neural development signals were mainly detected at 16 years and above, sex differentiation between 12 and 14 years, immunoregulatory mechanisms between 19 and 20 years, and musculoskeletal development in the range from 12 to 19 years.

**FIGURE 5:**
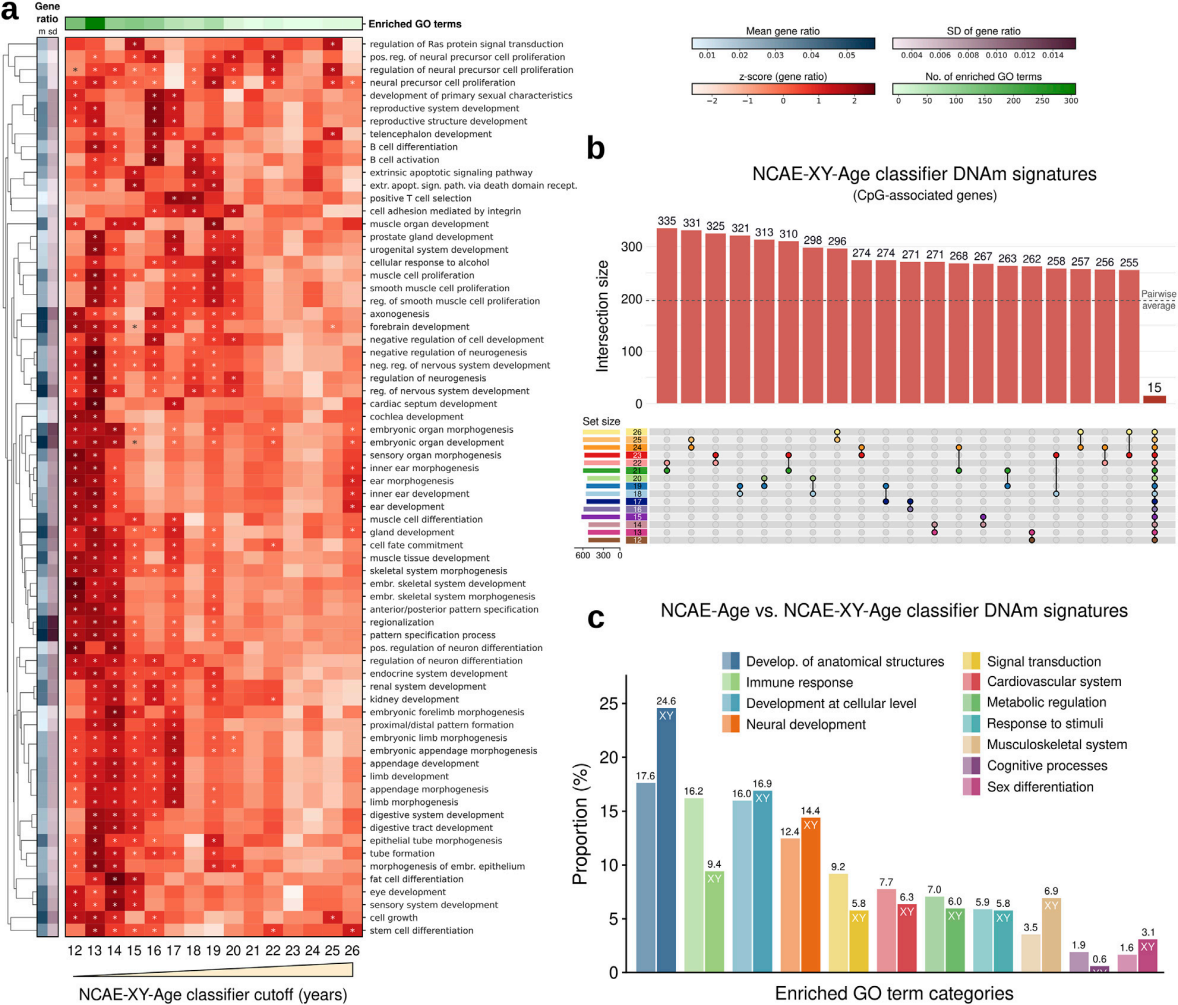
Functional enrichment analysis of CpG sites from NCAE-XY-Age DNAm signature genes. **(A)** Heatmap of gene ratio z-scores of the top enriched GO terms for age-specific DNAm signatures of aging from NCAE-XY-Age classifiers with cutoffs between 12 and 26 years. Significantly enriched (FDR-adj. p < 0.05) GO terms are marked with an asterisk (*). **(B)** Upset plot of non-exclusive pairwise overlaps between CpG-associated genes from NCAE-XY-Age classifier signatures of aging (12–26 years). **(C)** Bar plot of the proportion (%) of significantly enriched GO terms (n = 521) across NCAE-XY-Age DNAm signatures of aging and classifier cutoffs grouped into common GO ancestor categories compared with NCAE-Age signatures.

To account for sex-specific differences that could affect developmental changes during youth, we next trained a variant of NCAE-Age clocks including an additional 10,619 CpG sites from the X and Y chromosomes, named NCAE-XY-Age models (Supplementary Material S2). These clocks achieved similar benchmark results as NCAE-Age models in whole blood (R^2^ = 0.958, MAE = 3.12 years, and RMSE = 4.67 years) (Supplementary Material S3) and in other tissues (Supplementary Table S2). Interestingly, their DNAm signature of aging was significantly enriched in 113 GO terms, which is higher than any other signature (Supplementary Figure S1; Supplementary Material S7). We proceeded by training fifteen young-age NCAE-XY-Age classifiers with cutoffs from 12 to 26 years and retrieving their single-age aging signatures (Figures 5A, B; Supplementary Material S6). We found 521 significantly enriched GO terms across signatures, out of which 172 overlapped with the 426 enriched terms from NCAE-Age (Fisher’s exact test P = 1.61e-82) (Supplementary Material S7). Compared with NCAE-Age results (Figure 5C), XY signatures showed a higher proportion of biological processes associated with the general development of anatomical structures (128/521; 24.6% vs 17.6%), the musculoskeletal system (36/521; 6.9% vs 3.5%), and sex differentiation processes (16/521; 3.1% vs 1.6%). Notably, enriched GO terms for organismal and cellular development and sex differentiation were found mainly between 12 and 14 years. Immune-related terms were less common (49/521; 9.4%), and their enrichment levels, as well as those for neural (75/521; 14.4%) and musculoskeletal development, decreased with age (Supplementary Material S7). Other categories were approximately equally distributed across the age range.

### NCAE-Age clocks identify epigenetic signatures of delayed aging in pediatric Crohn’s disease

Lastly, we hypothesized that the effects of disease on normal development could be characterized by estimating age probabilities and identifying candidate underlying mechanisms using the DNAm signatures of aging. For that purpose, we chose to study the developmental consequences of Crohn’s disease (CD) in children and young adults. CD is a form of inflammatory bowel disease (IBD) that presents as an immunologically mediated, chronic remittent and relapsing inflammation of the gastrointestinal tract. Pediatric CD has an earlier onset of immunological disruptions and is associated with irregular disease progression (Van Limbergen et al., 2008; Ruemmele et al., 2014)⁠. Pertinent to our aim, between 65% and 85% of children and adolescents diagnosed with CD suffer from growth deficiency or delay (Gasparetto and Guariso, 2014; Wong et al., 2021; Rosen et al., 2015)⁠.

**FIGURE 6:**
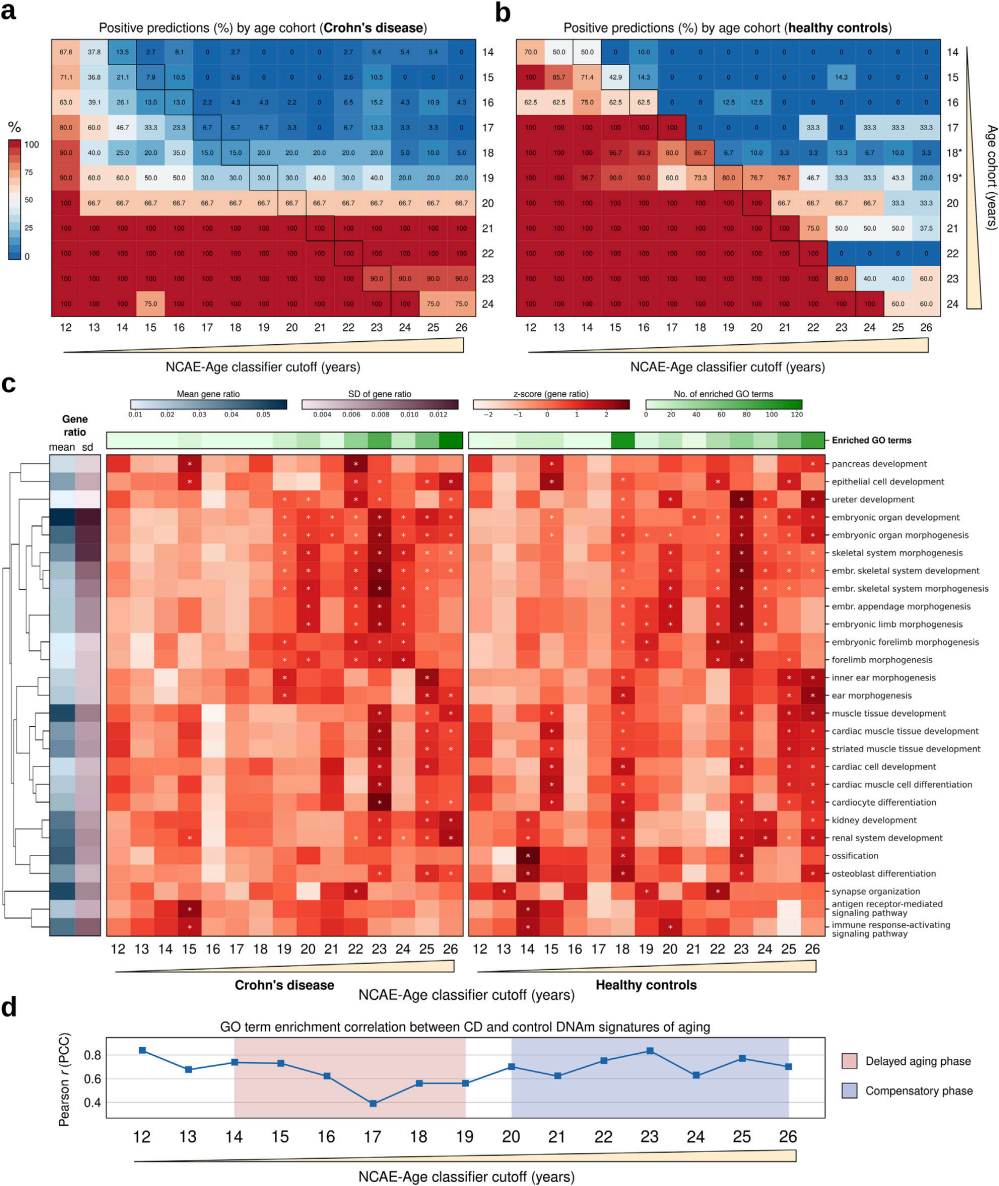
Epigenetic age and functional enrichment analysis of signatures of aging in Crohn’s disease (CD) in treatment-naive adolescents and young adults. **(A, B)** Heatmaps of positive predictions (%; estimated probability >0.5) per NCAE-Age classifier for single-year cohorts (14–24 years) of age-matched individuals with CD and healthy controls to be equal to or above the NCAE-Age classifier cutoffs (12–26 years). Outlined cells indicate that the true age of the single-age cohort individuals is equal to the NCAE-Age classifier cutoff. Cohorts include all available persons with CD and study-matched controls of the same age. Age-matched controls, who are 18 and 19 years old, from the classifier test set replace non-available controls from CD datasets. **(C)** Heatmaps of the gene ratio z-scores of the top enriched GO terms for DNAm signatures of aging from NCAE-Age classifiers with cutoffs between 12 and 26 years for CD patients (left) or controls (right). Significantly enriched (FDR-adj. *p* < 0.05) GO terms are marked with an asterisk (*). **(D)** Pearson correlation coefficients for GO term enrichment levels of CD and control DNAm signatures of aging across NCAE-Age classifier cutoffs. Delayed aging and compensatory (accelerated) aging phases are highlighted.

We gathered publicly available whole-blood DNAm datasets comprising CD patients and healthy controls aged between 14 and 24 years. The final cohort consisted of 207 CD patients (mean ± SD age = 17.1 ± 2.5 years) and 52 healthy controls (mean ± SD age = 18.6 ± 3.5 years) (Supplementary Material S1). All CD samples were reportedly collected at the time of disease detection from treatment-naive individuals, thereby eliminating potential confounding effects of medical treatments. To assess biological aging, we generated age probability estimates for each sample using the NCAE-Age classifier array, applying age cutoffs ranging from 12 to 26 years. We compared the proportion of positive predictions (i.e., predictions above the true age) below and above each cutoff between CD patients and controls (Figures 6A, B; Supplementary Material S4). Interestingly, we observed that CD patients with ages from 14 to 19 years had their true age consistently underestimated by NCAE-Age classifiers (Figure 6A). For instance, only 7.9% of the 15-year-old and 20.0% of the 18-year-old CD patients were predicted to be 15 years or older. In contrast, 42.9% of 15-year-old and 96.7% of 18-year-old healthy controls were accurately predicted to be 15 years or older. Similarly, only 15.0% of 18-year-old CD patients were predicted to be 18 years or older, compared to 86.7% of 18-year-old controls. Yet, between 66.7% and 100% of CD patients from 20 to 24 years were estimated to be above 26 years, compared to between 33.3% and 60.0% of controls in the same age range. These findings suggest that CD patients experience a delayed pace of biological aging in their teenage years, which reverses after the age of 20, leading to accelerated aging in early adulthood. Healthy controls did not exhibit accelerated or decelerated aging patterns at any age (Figure 6B), aligning with previous results observed in control test sets (Figure 3F). No significant differences in the epigenetic aging of CD patients due to sex were observed (Supplementary Figure S2).

To characterize this diverging pace of aging in CD, we retrieved age-specific DNAm signatures and explored their enrichment in biological processes represented by GO terms (Figure 6C; Supplementary Material S7). We found 271 significantly enriched unique GO biological processes (FDR-adj. P < 0.05) in the signatures of aging from NCAE-Age classifiers (12- to 26-year cutoffs) from CD patients and controls, including every GO term with at least one significant hit in at least one signature. The most represented term categories were the development of anatomical structures (27.3%; 74/271), developmental processes at the cellular level (17.3%; 47/271), neural system development (13.7%; 37/271), and musculoskeletal system development (11.1%; 30/271) (Supplementary Material S7). Enrichment levels from CD patient and control signatures across the measured age range were moderately correlated (PCC = 0.67). However, restricting the analysis to the interval when delayed aging in CD was observed (14–19 years) resulted in a gradual decrease in the correlation between enrichments of CD patients and control signatures, with the largest discrepancy being observed at 17 years (PCC = 0.39) (Figure 6D). For this interval, control signatures had a total of 139 significant hits for enriched GO terms, while CD signatures had only 19 hits. Significant terms for controls included the following: multicellular organism growth, muscle tissue development, peripheral nervous system development, renal system development, ossification, bone mineralization, stem cell proliferation, and positive regulation of peptidase and endopeptidase activity, among others. None of these terms were significantly enriched in CD signatures until 19 years. The development of anatomical structures remained the largest category in both cases (37.2% for controls; 52.6% for CD). However, the proportion of enriched GO terms associated with the immune response was twice as high for CD signatures compared to controls (10.5% vs 5.0%). Other categories, such as the development of the musculoskeletal system (14.0% vs 15.8%) or the development of the neural system (7.4% vs 5.3%), were similarly represented. From 20 to 26 years, the GO enrichment levels of signatures from both sample groups gradually converged until reaching PCC *=* 0.70 at the 26-year classifier. CD signatures had a total of 354 significant hits for GO term enrichments, whereas control signatures had 264 hits. These results align with those of the previously observed delayed aging phase from 14 to 19 years and posterior compensatory accelerated aging for CD patients after the age of 20 years (Figure 6D; Supplementary Material S7).

## Discussion

In this study, we present NCAE-CombClock, a high-precision neural network model for age estimation trained on a combination of autoencoder embeddings from whole-blood DNAm data and established age clock CpG sites. NCAE-CombClock demonstrated exceptional accuracy in predicting chronological age across a large dataset (n > 17,000) encompassing the entire human lifespan (0–112 years), achieving an MAE of less than 2 years. While existing DNAm age clocks can accurately determine whether individuals fall below or above crucial young-age milestones (15, 18, and 21 years), their susceptibility to misclassification limits their practical utility. To address this, we also developed a suite of neural network classifiers (the NCAE-Age array) trained on DNAm embeddings to generate probability estimates of age at various cutoffs, facilitating the reliable classification of DNAm samples at the single-year level. Furthermore, these models are interpretable and can thus serve as exploratory tools for identifying data-driven DNAm signatures associated with aging. Functional enrichment analyses revealed that the NCAE-Age signatures retrieved from healthy adolescent and young adult cohorts between 14 and 24 years were significantly enriched in GO terms associated with developmental mechanisms, such as the development of anatomical structures, neural system maturation, immunoregulation, and metabolism.

The results of the regression benchmark demonstrated that limited sets of specific CpG sites from DNAm age clocks could predict chronological age with near-perfect precision, achieving test set R^2^ values between 0.897 and 0.969. Additional predictive performance was gained by an elastic net DNAm age clock utilizing the combined clock CpG sites (CombClock; R^2^ = 0.974). However, the fraction of non-stochastic CpG sites selected by clocks built via penalized regression could be as low as 10% of their total number of sites (Tong et al., 2024)⁠ and likely reflects genes involved in or affected by different age-related mechanisms spread throughout the interactome, rather than part of a localized biological process (Levine et al., 2022; Liu et al., 2020)⁠. This implies that a large portion of useful epigenetic markers, regardless of whether stochastic or not, are inevitably excluded. To leverage all available DNAm information efficiently and allow for downstream interpretability, we compressed the data into non-linear autoencoder embeddings, enhancing the capture of complex patterns that provide a data-rich environment for explainable models. This strategy resulted in optimized age estimation via the NCAE-CombClock, with a top R^2^ of 0.978, and interpretable single-year DNAm signatures of aging, as demonstrated with the NCAE-Age classifier array.

Adolescence and early adulthood are periods of rapid epigenetic change, influenced by both intrinsic developmental processes and extrinsic environmental factors (Simpkin et al., 2017; Jones et al., 2015)⁠. Some of the DNAm age clocks benchmarked in this study were developed specifically for age prediction in children and adolescents. For example, Li’s clock (Li et al., 2018)⁠, trained using DNAm samples from individuals between 6 and 17 years, expectedly performed best at the 15-year threshold. Likewise, training data for PAYA (Aanes et al., 2023)⁠ spanned 12–25 years, resulting in accurate classifications of 18-year-olds with relatively low FPR. NCAE-Age performed at the same or higher level than Li and PAYA clocks across every age cutoff, while CombClock (in young-age training setting) and NCAE-CombClock (complete-age training) were tied in F1-score at the 15-, 18-, and 21-year thresholds. Finally, the consensus of the top five clocks produced the highest F1-score and lowest FPRs across categories, underscoring the potential of ensemble-based approaches. Nevertheless, utilizing an array of clocks such as our introduced NCAE-Age classifiers offers several advantages over a single or a consensus of clocks for ensuring robust predictions in legal, clinical, and other real-life contexts. Probability estimates provide a clearer understanding of uncertainty and confidence levels for decision-makers, enabling a degree of redundancy that decreases the impact of outliers or errors from any one model. In the context of health, they may also help guide tailored preventive or therapeutic interventions and allow for risk stratification.

DNAm age clocks have been shown to be associated with aging factors and outcomes such as mortality, smoking, male sex, body mass index, cardiovascular disease, and cancer (Oblak et al., 2021)⁠. Our goal was to advance beyond merely investigating associations of predicted epigenetic age. Instead, we aimed to implement interpretable clocks that identify data-dependent DNAm signatures relevant to aging, thereby validating their reliability. The analysis of signatures retrieved from the NCAE-Age classifier array found multiple significantly enriched age-related biological processes. For example, regulation of organ growth, stem cell division and differentiation (Brunet et al., 2023)⁠, regulation of the Wnt signaling pathway (Nusse and Clevers, 2017)⁠, and Ras signal transduction with positive regulation of the ERK1 and ERK2 cascade (Slack et al., 2015)⁠. Several of these processes, especially those found in the NCAE-XY-Age signatures, were markedly linked to developmental events occurring during adolescence and young adulthood, such as the development of primary male sexual characteristics, forebrain development, and mammary gland epithelium development. This highlights the specificity of NCAE-Age DNAm signatures in reflecting youth-specific mechanisms.

Currently, clinical and lifestyle interventions aimed at mitigating senescence are usually carried out in older individuals from 50 to 70 years (Fahy et al., 2019; Fitzgerald et al., 2021; Fitzgerald et al., 2023; Cano-Ibáñez and Bueno-Cavanillas, 2024)⁠. However, emerging evidence suggests that interventions during earlier life stages can significantly influence epigenetic aging trajectories (Mareckova et al., 2023; Harris et al., 2024)⁠. NCAE-Age signatures of epigenetic aging could be used alongside personalized long-term healthcare strategies starting at a younger age, enabling researchers to measure the longitudinal impact of lifestyle factors such as diet, physical activity, stress management, or exposure to environmental toxins on DNAm patterns over time. Individuals affected by age-related diseases could also benefit from specifically tailored interventions to modulate DNAm aging signatures and mitigate disease progression, thus improving health outcomes. For instance, anti-inflammatory diets or targeted pharmacological treatments could influence DNAm at CpG sites associated with disease mechanisms, as suggested by our NCAE-Age signatures.

In our analysis of pediatric CD, we demonstrated how the NCAE-Age classifier array can be effectively implemented in clinical settings to gain valuable insights into epigenetic aging processes, disease progression, and potential personalized therapeutic strategies. We identified a delayed pace of aging in CD patients between 14 and 19 years, which is consistent with existing literature (Gasparetto and Guariso, 2014; Wong et al., 2021; Rosen et al., 2015)⁠ reporting similar trends in epigenetic aging dynamics in CD. DNAm signatures from CD patients within this age range, obtained from the NCAE classifiers, exhibited enrichment for a small number of GO terms related to developmental processes. However, the extent of this enrichment was significantly lower compared to that observed in the control cohorts, suggesting that the epigenetic aging mechanisms in CD patients may diverge from typical developmental pathways, potentially reflecting disease-specific regulatory alterations. The aging pace in CD then accelerated until the last measured cutoff of 26 years, compensating for the prior delay through an overactivation of developmental processes, as evidenced by the functional enrichment of their DNAm signatures of aging after 20 years of age.

While our approach advances DNAm age estimation and explainability, it has certain limitations. The use of deep neural networks introduces a degree of complexity, requiring extensive training and hyperparameter optimization. Although the NCAE-Age classifiers provide high-resolution insights at young ages, a more robust interpretation of DNAm signatures of aging could be achieved by expanding the studied age range to below 12 and above 26 years. Employing shorter time scales, such as semesters or months, with appropriate training data could also increase result granularity. Most DNAm age clocks included are trained on whole-blood data, potentially missing epigenetic variations associated with aging in other tissues. Notably, NCAE-Age clocks perform accurately in whole blood, PBMCs, PBLs, saliva, and buccal epithelium. Therefore, conducting functional analyses of models trained on these tissues could further validate our findings. Finally, the cohorts available in public repositories predominantly consist of samples of Caucasian origin, which may hinder generalizability. Future work could focus on validating our findings with the collection of a more demographically and ethnically diverse body of data and explore the influence of clinical, lifestyle, and environmental factors on DNAm signatures of aging over longer time periods.

In conclusion, our study underscores that for age estimation models to be reliable, they must effectively capture age-related mechanisms within their DNAm signatures. We developed NCAE-CombClock, a novel and highly precise age prediction model leveraging DNAm embeddings and CpG sites. Additionally, we introduced a neural network classifier ensemble to enhance the precision of age classification in young individuals. By examining the DNAm signatures from these interpretable models, we identified key biological processes associated with age and development during youth. These findings provide valuable insights into the mechanisms of aging in adolescence and young adulthood and have important implications for improving health outcomes through earlier and more personalized interventions.

## Data Availability

The datasets presented in this study can be found in the Gene Expression Omnibus (GEO) online repository. The accession numbers can be found in the article and supplementary material. The trained elastic net and NCAE-based DNAm age clocks are available in Figshare (doi: 10.6084/m9.figshare.26369998 and doi: 10.6084/m9.figshare.26370106, respectively). The code used for data processing, model training, and signature analysis is available in Zenodo (doi: 10.5281/zenodo.12906211).

## Abbreviations

AE, autoencoder; CD, Crohn’s disease; CpG, 5′-cytosine-phosphate-guanine-3′ site; DNAm, DNA methylation; DNN, deep neural network; FDR, false discovery rate; FPR, false positive rate; GEO, Gene Expression Omnibus; GMQN, Gaussian mixture quantile normalization; GO, Gene Ontology; IBD, inflammatory bowel disease; NCAE, network-coherent autoencoder; NCBI, National Center for Biotechnology Information; PBL, peripheral blood lymphocyte; PBMC, peripheral blood mononuclear cell; ReLU, rectified linear unit; SNP, single-nucleotide polymorphism; WB, whole blood.
