# Supplementary figures and images for: Precise and interpretable neural networks reveal epigenetic signatures of aging across youth in health and disease
Source: Front Aging. 2025 Jan 23;5:1526146. doi: 10.3389/fragi.2024.1526146 (PMC11799293; doi:10.3389/fragi.2024.1526146)

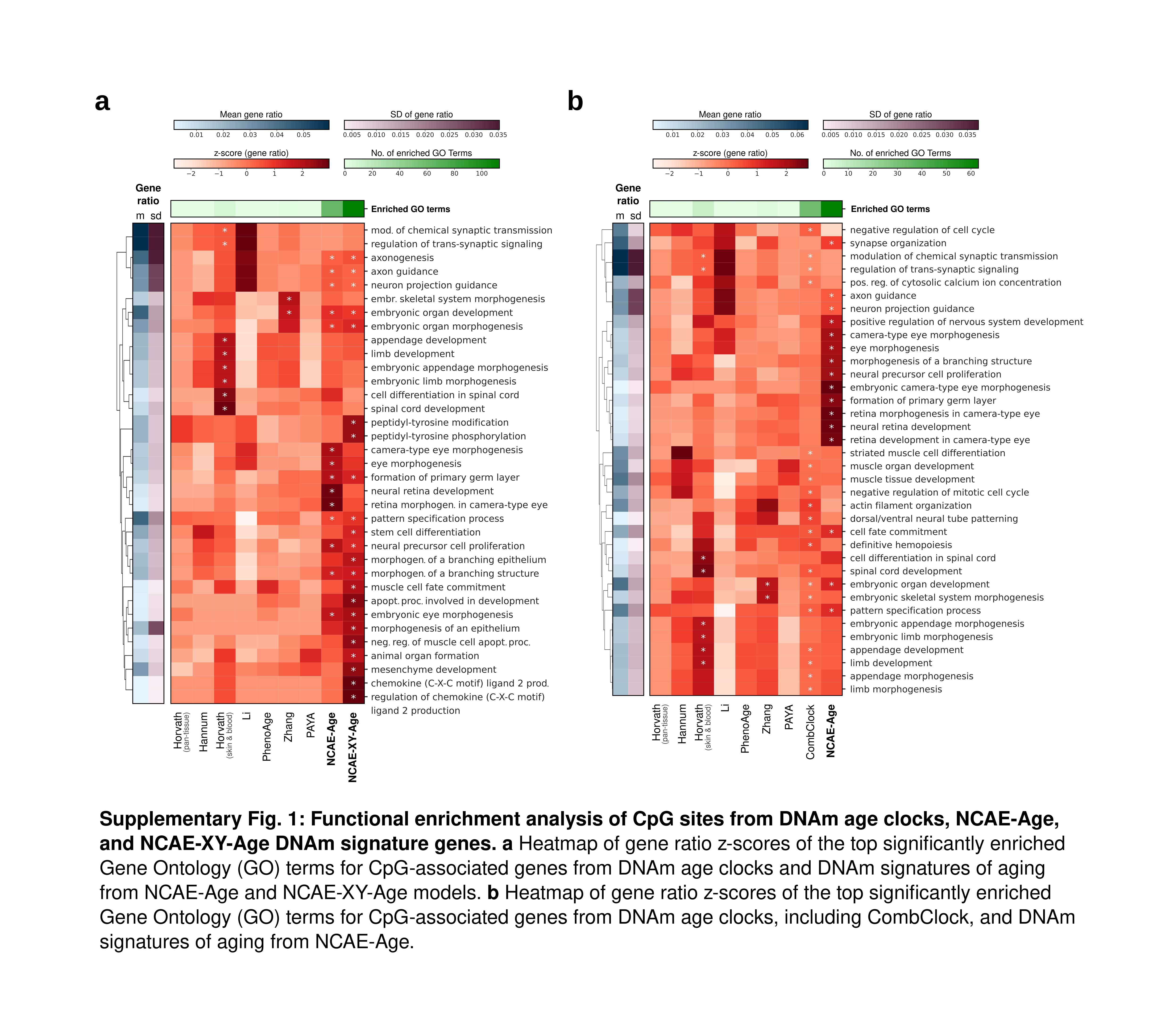

Supplement: Supplementary file 2 [file Image1.tiff]

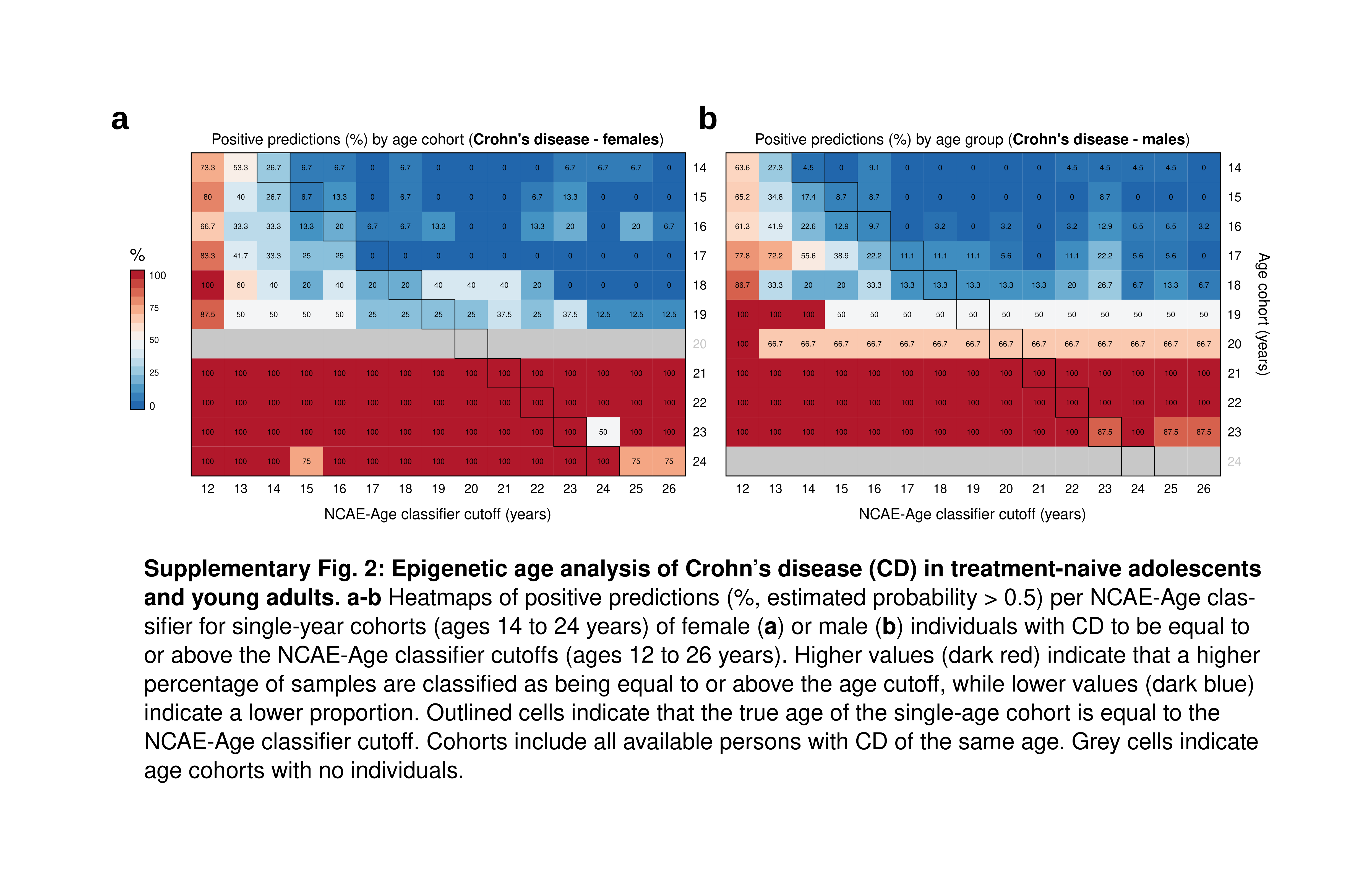

Supplement: Supplementary file 8 [file Image2.tiff]
